# Impact of Two Reoviruses and Their Coinfection on the Rice RNAi System and vsiRNA Production

**DOI:** 10.3390/v10110594

**Published:** 2018-10-30

**Authors:** Zhanbiao Li, Tong Zhang, Xiuqin Huang, Guohui Zhou

**Affiliations:** 1 Guangdong Province Key Laboratory of Microbial Signals and Disease Control, College of Agriculture, South China Agricultural University, Guangzhou 510642, China; lizhanbizo8410@sina.com (Z.L.); zhangtong@scau.edu.cn (T.Z.); huangxiuqin2007@163.com (X.H.); 2 Integrative Microbiology Research Center, South China Agricultural University, Guangzhou 510642, China

**Keywords:** *southern rice black-streaked dwarf virus* (SRBSDV), *rice ragged stunt virus* (RRSV), *synergism*, RNA interference (RNAi), virus-derived interfering RNAs (vsiRNAs)

## Abstract

Both *Southern rice black-streaked dwarf virus* (SRBSDV) and *Rice ragged stunt virus* (RRSV) belong to the family *Reoviridae*, and synergistic infection of these two viruses commonly occurs in the field. This study revealed that both SRBSDV and RRSV affect the RNA interference (RNAi) pathway and form different virus-derived interfering RNA (vsiRNA) profiles in rice. Co-infection of rice by SRBSDV and RRSV up-regulated the expression of rice DICER-like (DCL) proteins but down-regulated the expression of rice RNA-dependent RNA polymerases (RDRs), and the accumulation of vsiRNAs of either RBSDV or RRSV was decreased compared with that in singly infected plants. The majority of SRBSDV vsiRNAs were 21 nt or 22 nt in length, whether plants were singly infected with SRBSDV or co-infected with RRSV. On the other hand, the majority of RRSV vsiRNAs were 20 nt, 21 nt, or 22 nt in length, among which those 20 nt in length accounted for the largest proportion; co-infection with SRBSDV further increased the proportion of 20 nt vsiRNAs and decreased the proportion of 21 nt vsiRNAs. Co-infection had no effects on the strand favoritism and hot spots of the vsiRNAs, but changed the bias of the 5′ terminal nucleotide significantly. This study provides a reference for further study on the pathogenesis and synergistic mechanism of SRBSDV and RRSV.

## 1. Introduction

RNA silencing (also called RNA interference (RNAi)) in plants can function as an antiviral defense mechanism against invading viruses. To counteract RNA silencing, many plant viruses have evolved viral suppressors of RNA silencing (VSR) that target various components of the plant RNAi machinery. Due to the effects of different viruses on RNAi components, plants display different symptoms and produce different profiles of virus-derived interfering RNAs (vsiRNAs) [1,2,3]. Until now, many reports have focused on the effects on the RNAi pathway and the characteristics of vsiRNAs during infection with a single virus. The effects of co-infection by two or more viruses are still rarely studied, though such co-infection commonly occurs under field conditions. Previous studies revealed that co-infection of maize by *Maize chlorotic mottle virus* (MCMV) and *Sugarcane mosaic virus* (SCMV) or co-infection of tobacco by Brassica yellows virus (BrYV, a provisional name) and *Pea enation mosaic virus 2* (PEMV 2) increased the accumulation of both of the two viruses’ vsiRNAs compared with that seen in individual infections of the viruses. This indicates that the effects on the RNAi pathway may differ between co-infection and individual infection by the viruses, and may play an important role in the viruses’ synergism [4,5].

Plants encode multiple DICER-like (DCL) proteins, Argonaute (AGO) proteins, and RNA-dependent RNA polymerases (RDRs) involved in antiviral defense [6,7]. DCLs cleave RNA molecules with double-stranded features, producing primary small interfering RNAs (siRNAs), and serve a function in antiviral defense. In *Arabidopsis thaliana*, AtDCL4 is responsible for the biogenesis of 21 nt vsiRNAs and plays an important role in defense against positive-stranded RNA viruses of plants. In the absence of AtDCL4, AtDCL2 can produce 22 nt vsiRNAs as a surrogate and contributes to antiviral defense. AtDCL3 plays a minor role in defense against RNA viruses, but the 24 nt vsiRNAs which are generated by AtDCL3 are involved in antiviral defense against DNA viruses [8,9]. AtDCL1 may act as a negative regulator limiting AtDCL4 and AtDCL3 through the miRNA pathway [10,11]. RDRs amplify single-stranded RNA (ssRNA) to generate secondary siRNAs. In *Arabidopsis thaliana*, AtRDR1, AtRDR2 and AtRDR6 serve important functions in secondary siRNA production of *Potato virus X* (PVX), *Cucumber mosaic virus* (CMV), *Tabacco mosaic virus* (TMV), *Sugarcane mosaic virus* (SCMV), *Turnip mosaic virus* (TuMV), and *Tobacco rattle virus* (TRV) [10,12,13,14,15]. In rice, OsRDR6 has been shown to be involved in resistance to *Rice stripe virus* (RSV) and *Rice dwarf Phytoreovirus* (RDV), but the function of other RDRs is still obscure [16,17]. Different AGO proteins recruit vsiRNAs with different lengths and 5′ terminal nucleotides to form RNA-induced silencing complex (RISC), then cleave viral RNA in a sequence-specific manner. In Arabidopsis, AtAGO1 was proved to be a main effector against *Brome mosaic virus* (BMV), CMV, *Turnip crinkle virus* (TCV), and TuMV [18,19,20], and AtAGO2 was proved to be a main effector against CMV, TCV, and PVX [21,22,23]. CMV 2b protein (silencing suppressor protein) directly interacts with AtAGO4 and counteracts AtAGO4-related functions, thus creating a favorable cellular niche for CMV proliferation [24]. AtAGO5 was shown to be a main effector of defense against PVX and AtAGO7, and as a surrogate of AtAGO1, it contributes to defense against TCV [10,21]. There are 19 OsAGOs in rice [25], and little is known about their function. Only OsAGO1 and OsAGO18 have been found to be main effectors of defense against RDV and RSV, OsAGO18 sequesters microRNA168 (miR168) to increase the accumulation of OsAGO1 to confer broad-spectrum virus resistance in rice [26].

Different virus–plant combinations may form vsiRNAs with different characteristics, leading to different symptoms in plants. The profiles of vsiRNAs have been found to change in different plants infected with the same virus: the characteristics of *Tomato spotted wilt virus* (TSWV)-derived small interfering RNA in TSWV-infected *Solanum lycopersicum* and *Nicotiana benthamiana* were significantly different [27], and Tospovirus-*Polygonum ringspot virus* (PolRSV)-infected *Solanum lycopersicum* and *Nicotiana benthamiana* showed similar results [28]. vsiRNA profiles differed in *Rice black-streaked dwarf virus* (RBSDV)-infected rice and RBSDV-infected maize, with the 21 nt class most prevalent in RBSDV-infected rice but the 22 nt class most prevalent in RBSDV-infected maize [29,30]. The profiles of vsiRNAs have also been shown to have different characteristics in the same species of plant infected with different virus strains: vsiRNA profiles varied among potato infected with different PVY strains, probably due to the different responses to RNA silencing [31].

*Southern rice black-streaked dwarf virus* (SRBSDV) and *Rice ragged stunt virus* (RRSV) are species of the genera *Fijivirus* and *Oryzavirus*, respectively, in the family *Reoviridae*. The two viruses are transmitted by long-distance migratory pests, the white backed planthopper (WBPH, *Sogatella furcifera*) and the brown rice planthopper (BPHs, *Nilaparvata lugens*), respectively, in a persistent, circulative, and propagative manner [32,33]. Both viruses are widespread in the rice production regions of southern and southeastern Asia, and cause severe rice yield loss. The genomes of SRBSDV and RRSV both include 10 linear double-stranded RNAs. SRBSDV encodes six putative structural proteins (P1, P2, P3, P4, P8, P10) and seven putative nonstructural proteins (P5-1, P5-2, P6, P7-1, P7-2, P9-1, P9-2); among them, P6 functions as a VSR and P10 as a major outer capsid protein [32]. RRSV encodes 11 proteins, and S6 and S10 encode the two nonstructural proteins Psn 6 and Psn 10, respectively. Psn 6 functions as a VSR and Psn10 is part of the viromatrix [34,35]. Rice plants singly infected with SRBSDV or RRSV exhibit dwarfism and dark green leaves; co-infection of rice by these two viruses increases the severity of these symptoms, but no new symptoms appear [36]. In co-infected rice plants, the accumulation of both viruses is significantly increased, and the efficiency of virus acquisition by insect vectors from co-infected plants increased. All of these results indicate the synergism of the two viruses [36,37]. So far, several studies have reported the effect of the RNAi pathway and the vsiRNA profiles in SRBSDV singly infected rice plants [38], but none has yet reported the same in RRSV or RRSV co-infected with SRBSDV. In the present study, high-throughput sequencing and Reverse transcription quantitative polymerase chain reaction (RT-qPCR) were used to study the characteristics of vsiRNAs and the expression of the core proteins of the RNAi pathway in co-infected rice leaves. The results will favor understanding of the synergism mechanism between SRBSDV and RRSV.

## 2. Materials and Methods

### 2.1. Plant and Virus Materials

The seeds of the ‘*Nipponbare*’ rice cultivar used in this research were maintained in our laboratory. Plant culture and virus inoculation followed that described by Li et al. [37] with minor revisions. Briefly, the seeds were germinated by soaking in warm water, then sown in a plastic box with soil–matrix (soil:matrix = 1:1), where they were maintained until the third-leaf stage. A number of uniform seedlings of third-leafstage were selected and individually transferred into culture tubes (30 plants for each treatment). Five late-stage WBPH or BPH nymphs were used to inoculate the rice plants; the plants singly infected with SRBSDV or RRSV were obtained by inoculation with viruliferous WBPHs or BPHs, respectively, and the plants co-infected with SRBSDV and RRSV were obtained by inoculation with both viruliferous WBPHs and BPHs. Rice seedlings that had been inoculated with the same number of virus-free vectors (mock inoculated) served as healthy rice controls. All rice plants were transplanted in the greenhouse for observation of symptoms and sample collection.

### 2.2. RNA Extraction and Virus Detection

Total RNA from leaves of the rice plants was extracted using RNA isolater Total RNA Extraction Reagent as instructed by the manufacturer (Vazyme Biotech Co., Nanjing, China), and the presence of viruses in each inoculated plant was confirmed by RT-PCR at 10 days post inoculation (dpi) using a method previously described [39]. 

### 2.3. Small RNA Sequencing and Analysis

Leaf samples from rice plants infected with SRBSDV, RRSV, and SRBSDV+RRSV were harvested at 15 dpi. Samples from ten rice plants of the same infection status were collected together to form a pool of samples for RNA extraction using RNA isolater Total RNA Extraction Reagent (Vazyme Biotech Co., Nanjing, China). RNA products were treated with DNase I (TaKaRa, Dalian, China) and their concentration, integrity, and purity were verified by agarose gel electrophoresis and an Agilent Bioanalyzer 2100 system (Agilent, Palo Alto, CA, USA). Deep sequencing of small RNAs was performed using an Illumina HiSeqTM 2500 platform (Novogen, Beijing, China). Briefly, sRNA libraries were produced as follows: Small RNAs (sRNAs) with size from 18 to 30 nt were recovered by 15% denaturing polyacrylamide gel electrophoresis of total RNA, ligated with RNA adaptors (both the 5′ terminal and the 3′ terminal), and then reversely transcribed into cDNAs. These cDNAs were amplified by PCR, and the PCR products were recovered and enriched. The quality of the libraries were qualified using an Agilent 2100 Bioanalyzer (Agilent, Palo Alto, CA, USA) and a StepOnePlus Real-Time PCR System (Applied Biosystems), then subjected to Illumina HiSeqTM 2500 sequencing. Small RNA reads were quality-filtered to remove adapter sequences, low-quality reads, and reads smaller than 18 nt in length, and clean reads of small RNAs with size from 18 to 30 nt were extracted. To identify SRBSDV- and RRSV-specific small RNAs, these small RNA sequences were mapped to the SRBSDV genomic sequences (GenBank accession numbers:NC_014708~NC_014717) and RRSV genome sequences (GenBank accession numbers: NC_003749.1~NC_003752.1, NC_003757.1~NC_003759.1, NC_003769.1~NC_003771.1), respectively. Only the small RNAs matching viral sequences on the sense or antisense strands with at most 1 nt mismatch were recognized as vsiRNAs. 

### 2.4. Reverse Transcription Quantitative PCR

The second extensional leaves (the top leaf as the first leaf) of each infected plants were collected at 15 dpi. Total RNA from the leaves was extracted and purified, then the viral accumulation and the expression of proteins associated with the RNA silencing pathway were detected using RT-qPCR. The RT-qPCR assays were repeated three times, each with four biological replicates. The relative fold expression changes of target genes were calculated using the 2 delta-delta Ct method and *t*-test analysis or one way ANOVA analysis were conducted using SPSS19.0 software (IBM, New York, NY, USA) for windows. AceQ qPCR SYBR Green Master Mix (Vazyme Biotech Co., China) was used for RT-qPCR. The rice *OsEF-1a* gene was used as internal control. For all primers used in RT-qPCR, see Table S1 of the additional file.

## 3. Results

### 3.1. Co-Infection of SRBSDV and RRSV Exacerbated the Symptoms and Increased the Virus Titers

**Figure 1:**
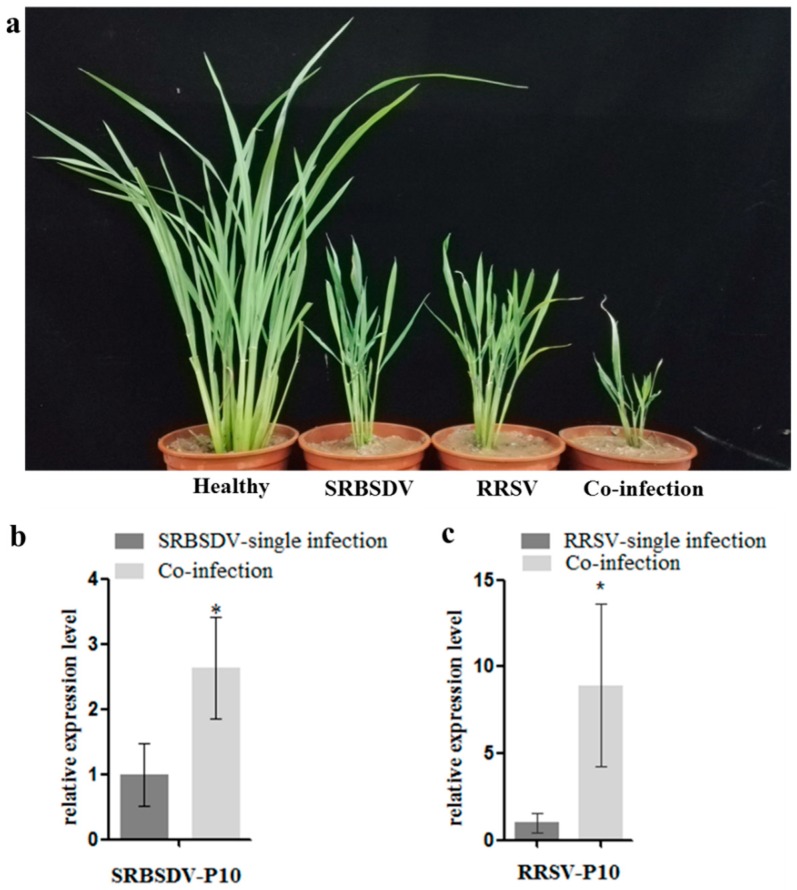
Co-infection of SRBSDV and RRSV exacerbates the symptoms (**a**) and increases the accumulation of the two viruses in rice plants (**b**,**c**). (**a**). Co-infection of SRBSDV and RRSV exacerbates symptoms (left to right: Healthy:Healthy rice plant; SRBSDV:SRBSDV singly infected plant, RRSV:RRSV singly infected plant; Co-infection: SRBSDV + RRSV); (**b**). Co-infection of SRBSDV and RRSV increased the accumulation of SRBSDV P10 mRNA; (**c**). Co-infection of SRBSDV and RRSV increased the accumulation of RRSV P10 mRNA (Asterisk indicates significant differences (*p* < 0.05)).

To confirm the synergism of SRBSDV and RRSV, third-stage rice seedlings were inoculated with viruliferous WBPHs and/or BPHs; consequently, rice plants singly infected with SRBSDV or RRSV or doubly infected with SRBSDV and RRSV were obtained. Symptoms were observed at 40 dpi, and the co-infected (SRBSDV+RRSV) plants showed severe symptoms including extreme dwarfism and curling and ragged leaves (Figure 1a). The accumulation of both viruses was detected using RT-qPCR at 15 dpi; the results showed that the accumulation of SRBSDV p10 mRNA in co-infected rice plants was 2.6-fold higher than in singly infected plants, and that of RRSV p10 mRNA in doubly infected plants was 8.9-fold higher than in singly infected plants. These results support the previous reports and indicate the strong synergism between SRBSDV and RRSV.

### 3.2. Co-Infection of SRBSDV and RRSV Altered Virus-Induced RNAi Pathway Genes

DCLs, AGOs, and RDRs are core elements of the RNAi pathway and contribute to plant antiviral defense. To analyze the effect of viral infection on these proteins, the expression levels of parts of DCLs, AGOs, RDRs were characterized using RT-qPCR in both singly infected and doubly infected plants. 

**Figure 2:**
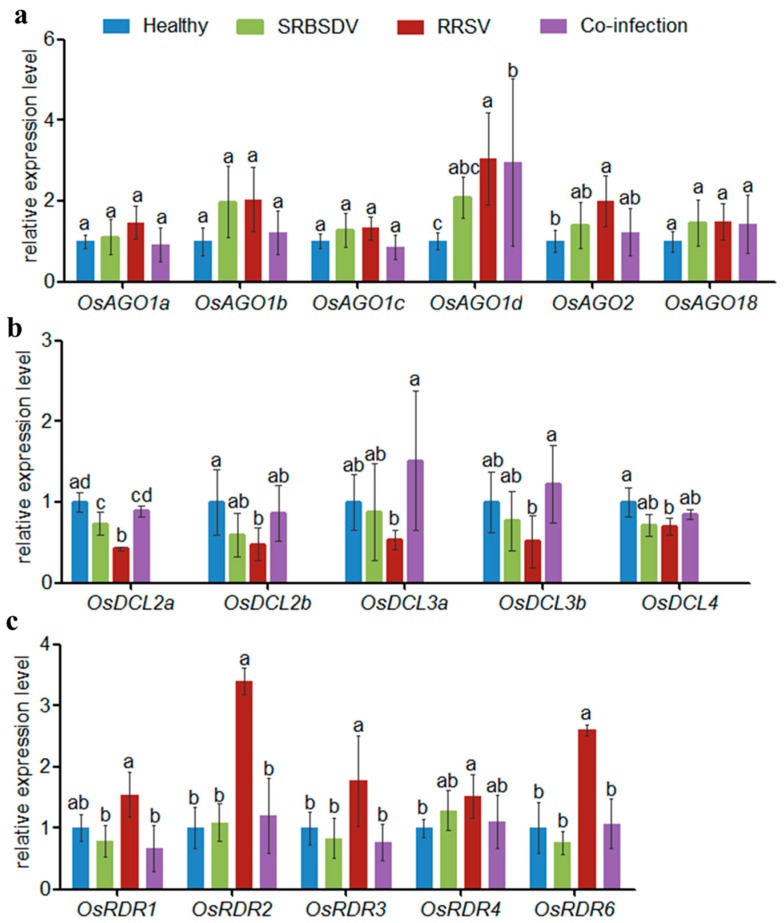
The relative expression of RNAi-associated proteins in singly and doubly infected plants as evaluated using RT-qPCR (**a**). The relative expression of AGOs in singly and doubly infected plants as evaluated using RT-qPCR (**b**). The relative expression of RDRs in singly and doubly infected plants as evaluated using RT-qPCR (**c**). The relative expression of DCLs in singly and doubly infected plants as evaluated using RT-qPCR. Lowercase letters indicate significant differences (*p* < 0.05).

The results showed that AGOs were mostly up-regulated whether in SRBSDV singly infected plants, RRSV singly infected plants, or doubly infected plants compared with healthy plants (Figure 2a). However, in doubly infected plants, only the expression levels of AGO1d showed significant difference with that in SRBSDV singly infected plants, by up-regulated 42.38%. Other AGOs showed no significant difference between singly and doubly infected plants, although the expression levels of AGO1a, AGO1b, AGO1c, AGO2 were down-regulated slightly in doubly infected plants compared with that in singly infected plants (Figure 2a).

All of the DCLs were down-regulated in SRBSDV and RRSV singly infected plants, but more so in RRSV singly infected plants compared with healthy plants (Figure 2b). In doubly infected plants, the expression levels of DCLs were up-regulated compared to those in singly infected plants, and DCL2a, DCL3a and DCL3b showed significant difference with that in RRSV singly infected plants, by up-regulated 108.97%, 181.75% and 136.00%, respectively. The expression of all DCLs showed no significant difference between SRBSDV singly infected plants and doubly infected plants.

In SRBSDV singly infected plants, RDR1, RDR3, and RDR6 were down-regulated but RDR4 was up-regulated slightly. In RRSV singly infected plants, all RDRs were up-regulated (Figure 2c). RDR1, RDR2, RDR3, RDR4, and RDR6 were down-regulated 131.44%, 183.60%, 130.96%, 37.45% and 144.75%, respectively, in doubly infected plants compared with in RRSV singly infected plants. However, no significant differences were found in RDR levels between SRBSDV singly and doubly infected plants.

### 3.3. Co-Infection of SRBSDV and RRSV Altered Virus-Induced RNAi Pathway Genes

Totals of 31,080,566 reads, 31,970,208 reads, and 28,219,238 reads of raw data (GEO NO.: GSE120754) were obtained from the small RNA library of SRBSDV singly infected plants, RRSV singly infected plants, and doubly infected plants (SRBSDV + RRSV), respectively (Table 1). From these totals, 27,486,888 reads (single SRBSDV), 27,299,736 reads (single RRSV), and 24,177,550 reads (SRBSDV+RRSV) of clean data were obtained by removing the low-quality reads, poly(A), and adaptor sequences. Reads ranging from 18 to 30 nt were mapped to the viral genomes to within 1 mismatch in both the sense and antisense orientations. The results showed that the vsiRNAs from SRBSDV singly infected plants (474,032 reads) were significantly fewer than vsiRNAs from RRSV singly infected plants (1,110,229 reads). However, in doubly infected plants, both the vsiRNAs from RRSV (346,402 reads) and the vsiRNAs from SRBSDV (69,192 reads) were fewer than the vsiRNAs from RRSV singly infected (1,110,229 reads) or SRBSDV singly infected plants (474,320 reads)—3.18-fold and 6.86-fold decreases in number, respectively (Table 1).

### 3.4. Co-Infection Changed the Length Distribution of the vsiRNAs of RRSV

**Figure 3:**
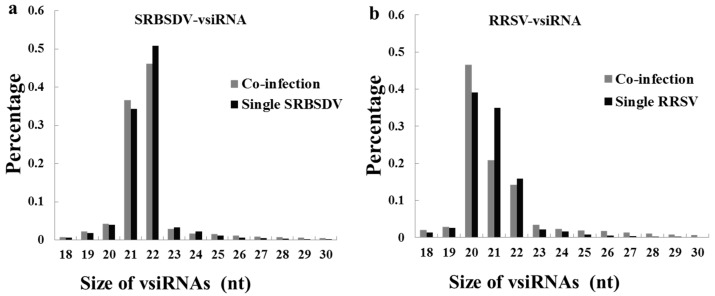
Size distribution of SRBSDV-derived small RNAs (**a**) and RRSV-derived small RNAs (**b**). (**a**) Size distribution of SRBSDV-vsiRNAs infected with SRBSDV or co-infected (SRBSDV + RRSV). (**b**) Size distribution of RRSV-vsiRNAs infected with RRSV or co-infected (SRBSDV + RRSV).

Both in singly and doubly infected plants, there are significant differences in the length distributions of the vsiRNAs between those from RRSV and those from SRBSDV. The majority of SRBSDV-vsiRNAs were 21 nt (SRBSDV single infection: 36.62%; CO-infection 34.24%) and 22 nt (SRBSDV single infection: 46.17%; CO-infection 50.86%) in length in both SRBSDV singly infected and doubly infected plants (Figure 3a); however, RRSV-vsiRNAs were 20 nt (RRSV single infection: 39.08%; CO-infection 46.52%), 21 nt (RRSV single infection: 34.89%; CO-infection 20.92%), and 22 nt (RRSV single infection 15.83%; CO-infection 14.26%) (Figure 3b). Interestingly, co-infection had no effect on the length distribution of SRBSDV-vsiRNAs, but had a significant effect on RRSV-vsiRNAs. The percentage of 20 nt RRSV-vsiRNAs in doubly infected plants increased 7.44% compared with in RRSV singly infected plants (46.52% in doubly and 39.08% in RRSV singly), but the percentage of 21 nt RRSV-vsiRNAs decreased 13.97% (20.09% in doubly and 34.89% in RRSV singly). To some extent, 23–30 nt RRSV-vsiRNAs were increased.

### 3.5. Co-Infection of SRBSDV and RRSV Had No Effect on the Strand Polarity of the vsiRNAs

**Figure 4:**
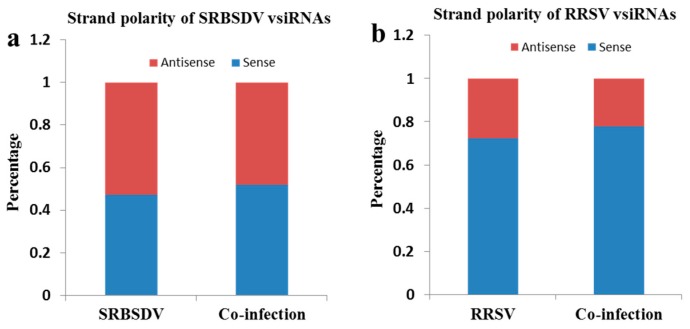
The strand polarity of SRBSDV-derived vsiRNAs (**a**) and RRSV-derived vsiRNAs (**b**). (**a**) Strand polarity of SRBSDV-vsiRNAs in SRBSDV- or SRBSDV + RRSV-infected plants. (**b**) Strand polarity of RRSV-vsiRNAs in RRSV- or SRBSDV + RRSV-infected plants.

To understand the origin of the vsiRNAs, the strand polarities of vsiRNAs were analyzed (Figure 4). There were no obvious differences in strand polarity for SRBSDV-vsiRNAs, either in SRBSDV singly infected or in doubly infected plants (Figure 4a), but a high prevalence of the sense (+) strand was observed in RRSV singly infected and doubly infected plants (Figure 4b). Despite the difference in the strand polarity of vsiRNAs between SRBSDV-vsiRNAs and RRSV-vsiRNAs, co-infection had no effect on the strand polarity for SRBSDV-vsiRNAs or RRSV-vsiRNAs. 

### 3.6. Co-Infection Changed the Bias of the 5′ Terminal Nucleotide of vsiRNAs

**Figure 5:**
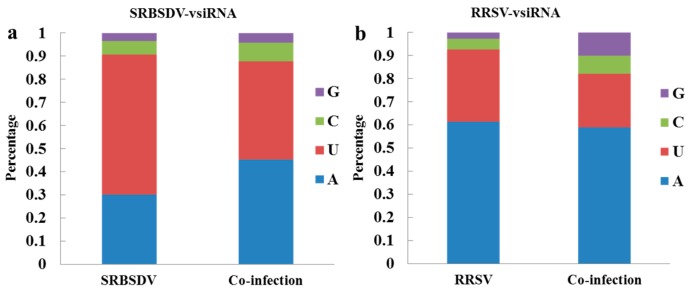
The proportions of 5′ terminal nucleotides of SRBSDV-derived vsiRNAs (**a**) and of 5′ terminal nucleotides of RRSV-derived vsiRNAs (**b**). (**a**) 5′-terminal nucleotides of SRBSDV-vsiRNAs in plants infected with SRBSDV or SRBSDV + RRSV. (**b**) 5′-terminal nucleotides of RRSV-vsiRNAs in plants infected with RRSV or SRBSDV + RRSV.

Previous studies have demonstrated that AGO proteins recruit small RNAs with different 5′-terminal nucleotides. To explore the potential interactions between small RNAs and AGOs, the 5′-terminal nucleotides of vsiRNAs were analyzed. A high prevalence of Uracil (U) and Adenine (A) for the 5′ terminal nucleotide was seen in singly (SRBSDV or RRSV) and doubly infected plants (SRBSDV + RRSV), and a correspondingly low percentage of Guanine (G) and Cytosine (C) (Figure 5). However, in doubly infected plants, the proportions of 5′ A and 5′ C among SRBSDV-vsiRNAs increased 15.18% and 1.91%, respectively, and that of 5′ U decreased 17.96% (Figure 5a). The proportion of 5′ U among RRSV-vsiRNAs decreased 8.34%, and those of 5′ C and 5′ G increased 3.27% and 7.32%, respectively, compared with those in singly infected plants (Figure 5b).

### 3.7. Co-Infection of SRBSDV and RRSV Had No Effect on vsiRNA Hotspots on the Viral Genome

All of the vsiRNA sequences were mapped along the sense and antisense strands of the SRBSDV and RRSV genomes, respectively. The maps indicated that vsiRNAs were almost continuously but heterogeneously distributed throughout the sense and antisense strands of SRBSDV and RRSV genomes in both singly and doubly infected plants (additional file1: Figure S1). Some hotspots were contained in the sense and antisense genomes. According to the maps, co-infection had no effects on the hotspot locations.

## 4. Discussion

Both SRBSDV and RRSV belong to the family *Reoviridae*, and cause severe rice yield loss in southern and southeastern Asia. A previous report showed that synergistic infection of SRBSDV and RRSV commonly occurs in the field [39]. In this study, we confirmed that co-infection of SRBSDV and RRSV produced severe symptoms in rice and increased the accumulation of both viruses in the rice plant; all of these results are consistent with typical synergistic symptoms. Synergistic infection of SRBSDV and RRSV increases the efficiency of virus acquisition by insect vectors from co-infected plants, thereby increasing the transmission of the viruses [36]. Studying the mechanism of synergistic infection of SRBSDV and RRSV will deepen knowledge of the behaviors of epidemics and provide possible avenues for disease control. This study explored the effects of the viruses on the RNAi pathway and the vsiRNA profiles both in SRBSDV or RRSV singly infected plants and in doubly infected plants.

Synergistic infection could increase vsiRNA accumulation of one or both viruses [4,5], but in this research, co-infection of SRBSDV and RRSV decreased the vsiRNA accumulation of both SRBSDV and RRSV (Table 1). RRS-vsiRNAs (3.18-fold less than when singly infected) decreased less than SRBSDV-vsiRNAs (6.86-fold less than when singly infected). A previous study demonstrated that interactions between synergistically interacting viruses can be asymmetric [40], so the interaction between SRBSDV and RRSV may also be asymmetric; future research is required to explore this possibility. The co-infection of SRBSDV and RRSV increased the accumulation of both SRBSDV and RRSV (Figure 1b,c) and the expression of DCLs (Figure 2b), which would lead to an increase in the accumulation of primary vsiRNAs. Therefore, the decrease in vsiRNA accumulation in doubly infected plants should be attributed to the decrease in secondary vsiRNA production caused by the decrease in RDR expression. Previous reports have shown that RDRs play an important role in the accumulation of vsiRNAs: CMV-derived vsiRNAs and TMV-derived vsiRNAs decreased significantly in AtRDR1 and AtRDR6 mutation plants, respectively, in Arabidopsis [14,15]; further, AtRDR1, AtRDR2, and AtRDR6 play important roles in the biogenesis of TRV- and TuMV-derived vsiRNAs [13,14]. In rice, the accumulation of RSV- and RDV-derived vsiRNAs decreased significantly in OsRDR6 mutation rice plants. Accordingly, we hypothesized that OsRDRs may play a role in the interaction between SRBSDV and RRSV in rice [16,17]. 

vsiRNAs originate from two sources: Highly structured positive viral RNA and double-stranded replication intermediates [41,42]. In this study, SRBSDV-derived vsiRNAs equally originate from the sense and antisense strands both in singly (SRBSDV) and in doubly infected plants (Figure 4a), consistent with previous reports [29,30,38]. The results indicate that the SRBSDV vsiRNAs originated from double-stranded replication intermediates. However, RRSV-derived vsiRNAs had a strong bias towards the sense strand whether in singly (RRSV) or doubly infected plants (Figure 4b), which indicated that RRSV vsiRNAs originate from highly structured positive viral RNA, and the increased accumulation of vsiRNAs from the sense strand is possibly due to the increase in the viral RNA replication. Both SRBSDV and RRSV belong to the family *Reoviridae*, but the origin of their vsiRNAs is different. Thus, this study provides new information on the origin of dsRNA plant-virus-derived siRNAs.

The 21 nt and 22 nt vsiRNAs play important roles in antiviral defense [2]. In this study, SRBSDV-derived vsiRNAs were mostly 21 nt or 22 nt in both singly (SRBSDV) and doubly (SRBSDV + RRSV) infected plants, which is consistent with previous reports [38,43], and co-infection had no obvious effects on their proportions. However, the majority of RRSV-derived vsiRNAs were 20 nt, 21 nt, or 22 nt; 20 nt accounted for the highest proportion, and co-infection increased the proportion of 20 nt. These results indicate that 20 nt vsiRNAs may play a role in RRSV–rice and RRSV–SRBSDV interactions. So far, the mechanism of the biogenesis and function of 20 nt small RNA remains elusive, though Xu et al. detected a large number of 20 nt vsiRNAs in RSV-infected rice and tobacco [44]. DCL4 and DCL2 were responsible for the production of 21 and 22 nt siRNA, respectively [45]. however, the expression levels of DCL4 and DCL2 showed no significant differences between singly and doubly infected plants (Figure 2b). In rice, DCL3a and DCL3b were shown to be responsible for the production of 24 nt siRNA [46,47]. In this study, the expression levels of DLC3a and DCL3b in doubly infected plants were higher than those in singly infected plants (Figure 2b), which led to an increase in the proportion of the 24 nt class. All results indicate that DCL3 may participate in the synergistic interaction between SRBSDV and RRSV.

AGOs recruit siRNAs with different 5′ terminal nucleotides to form AGO-containing RNA-induced silencing complexes (RISCs) to cleave target sequences and contribute to antiviral defense. AGO2/4 recruits siRNAs with 5′ A, and AGO1 recruits siRNAs with 5′ U [48,49,50]. AGO1 and AGO2 were identified as the major antiviral AGOs [48]. In this study, the majority of the 5’ terminal nucleotides were A and U, which indicates that AGO1 and AGO2 play an important role in virus defense against SRBSDV and RRSV. Notably, in co-infected plants, the proportion of SRBSDV-vsiRNAs with 5′ A increased, but RRSV-vsiRNAs showed no obvious changes. The proportions of both SRBSDV-vsiRNAs and RRSV-vsiRNAs with 5′ U decreased, which indicated that AGO2 may contribute in a major sense (compared with AGO1) to the synergistic interaction between SRBSDV and RRSV. The proportion of 5′ G and 5′ C was increased in the presence of both SRBSDV and RRSV, which indicates that other AGOs may play roles in the synergistic interaction between SRBSDV and RRSV [10,18,21,24].

## 5. Conclusions

This study confirmed that co-infection of SRBSDV and RRSV exacerbates the symptoms and increases the accumulation of both viruses, which is consistent with typical synergistic symptoms. The expression levels of some core elements of the RNAi pathway differed significantly in doubly infected plants compared with in singly infected plants: several DCLs were up-regulated and some RDRs were down-regulated, and these resulted to the decrease of the amount of vsiRNA from either SRBSDV or RRSV. Co-infection of SRBSDV and RRSV also altered the bias of the 5’ terminal nucleotide and the length distribution of vsiRNAs, but had no effects on strand favoritism or on hot spots of vsiRNAs. In addition, a large number of 20 nt long RRSV vsiRNAs were produced in RRSV singly infected plants and in those co-infected with SRBSDV, but little is known about their production and function. In summary, this study deepens our understanding of the synergism between SRBSDV and RRSV.

## Figures and Tables

**Table 1:** Classification and abundance of small RNAs from the SRBSDV, RRSV and SRBSDV+RRSV inoculated libraries.

Category	Reads		
	SRBSDV	RRSV	Co-Infection
Total raw reads	31,080,566	31,970,208	28,219,238
Total clean reads	27,486,888	27,299,736	24,177,550
SRBSDV-derived siRNAs	474,032	-	69,192
RRSV-derived siRNAs	-	1,110,229	346,402

## Supplementary Materials

The following are available online at http://www.mdpi.com/1999-4915/10/11/594/s1, Figure S1: Single-nucleotide resolution maps of SRBSDV-vsiRNAs and RRSV-vsiRNAs. Table S1: Primers used in this study.
