# Supplementary material for: Impact of Two Reoviruses and Their Coinfection on the Rice RNAi System and vsiRNA Production
Source: Viruses. 2018 Oct 30;10(11):594. doi: 10.3390/v10110594 (PMC6267445; doi:10.3390/v10110594)
Supplement: Supplementary file 1 [file viruses-10-00594-s001.pdf]

TableS1 Primers used in this study

Primer	Sequences（5'-3'）
**Primers used for quantitative RT-PCR**	
OsAGO1a-F1	GCCATTGGCGATCGACGAAG
OsAGO1a-R1	CCAACTGGAAGGCCATGTGC
OsAGO1b-F	TCTGTCGTTGATCCCGTTA
OsAGO1b-R	ACGCTCTGCAGCGTCACA
OsAGO1c-F	AGCTAGGGCCTACATAGC
OsAGO1c-R	TGGAGAAGATAGCAGCTG
OsAGO1d-F	GACTTCTGAAGCCACATGCT
OsAGO1d-R	GCCCAGCTTTTATGAAATAG
OsAGO2-F	AGACTCACAGGTTGTCTATCTTT
OsAGO2-R	CGAGAATTGCAAAATACAGAGT
OsAGO18-F	CGTTTCTACCTCACCCAA
OsAGO18-R	GCTCCAGGCTTCAGTTTC
OsDCL2a-F	GTCGTCGAGCAGCACACCG
OsDCL2a-R	GGCAGAGGGTCTGACGCACTAG
DCLs2b-F	CAAAGGCTGCTGAGGTACAATC
DCLs2b-R	GCTGTAGTTTCTAATGAAGCCCTC
OsDCL3a-F	ACGATTGCGATGCTTGACACAG
OsDCL3a-R	CTGTTGGTGCAAGAAATATGATGAC
DCL3b-F	CGTCCATCAGCAATTCGAGGTG
DCL3b-R	AACCCGCGATGCTCCAGAG
DCL4 qF1	GTACGCCAGCAAGCTGTGGTG
DCL4 qR1	CTCATGTCGTTCTCCCATTCCTG
RDR1 qF1	CCAAGGCCGCATCCTAATGAG
RDR1 qR1	GGTGTCACCATACGAGGCGG
OsRDR2-F	TGGACCTGTACCTGGAGCACG
OsRDR2-R	AGAGCAATACGGTCGGCAGC
OsRDR3-F	GCAACGACGCCAGCAGGTG
OsRDR3-R	GCGGTGTCACCCTCGTTCTTG
RDR4 qF1	TGAATCCGAGCTTGAAAGAGTGC
RDR4 qR1	CGCGCATAACTAGGTGCAAATC
OsRDR6-F	CAAGTGTTCATACGACGCGCAGC
OsRDR6-R	GTCGCCTTGCTTCTTGCTGCTG
rtRRSVs10-F	AGCCCGAGCCGCCATCATAG
rtRRSVs10-R	TAGCATCGCGCATCATCCC
rtSRBSDVs10-F	CGTGAAGTTCCTGCTCAAATGGG
rtSRBSDVs10-R	TTGGTCGTAACCGCCATAGTGTG
OsEF1a-F	ACATTGCCGTCAAGTTTGCTG
OsEF1a-R	AACAGCCACCGTTTGCCTC

Primers used for virus detection*	
SRB-S10-F	TGTTGTCGTGAAGTTCCTGCTC
SRB-S10-R	GGTCGTAACCGCCATAGTGTGTC
RRSV-CP-F	GCGTTCACAGCCGTTCAGTTTG
RRSV-CP-R	GCTTACCTGTGGCGTCAACCTC

*Yang et al.,2017

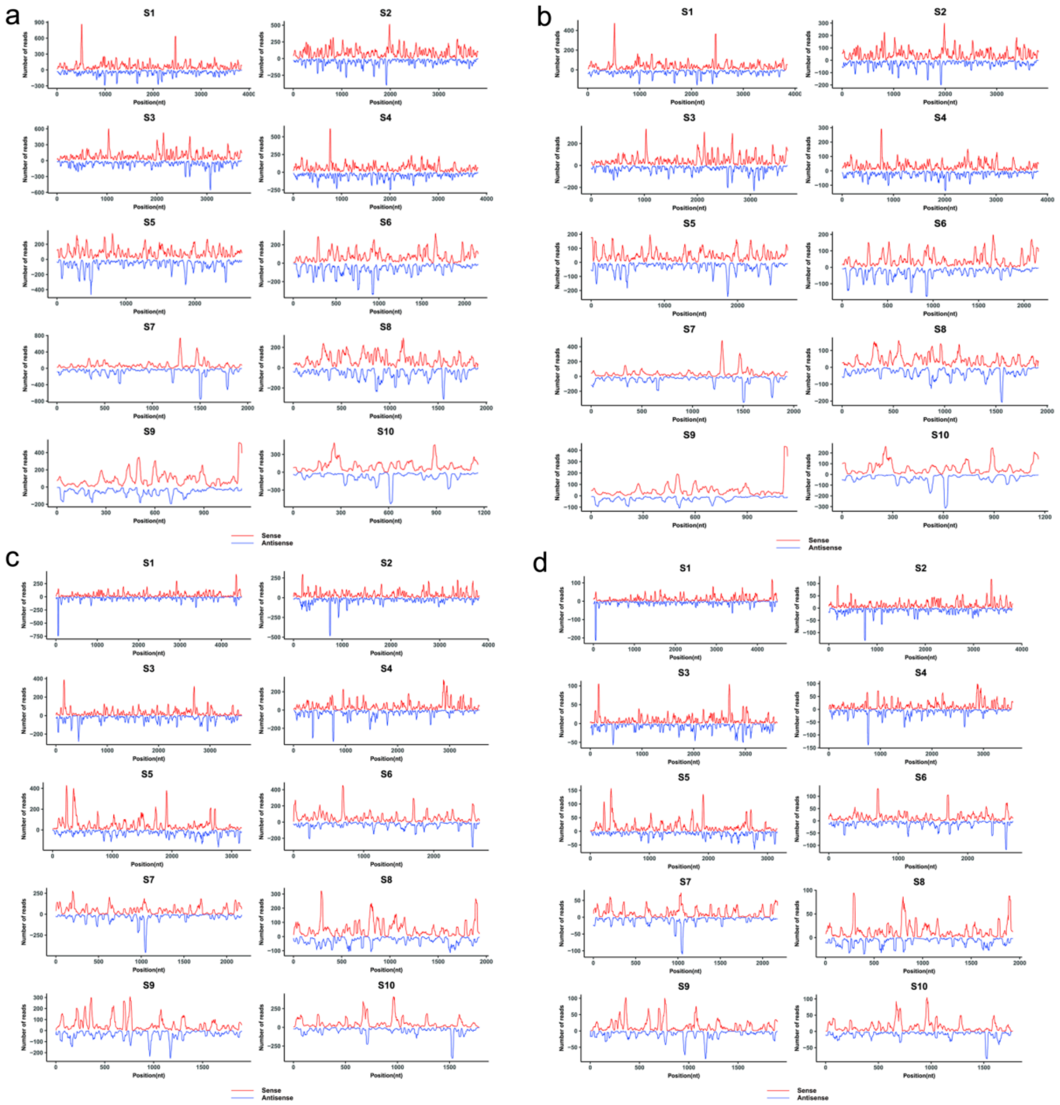

Fig. S1 Single-nucleotide resolution maps of SRBSDV-vsiRNAs and RRSV-vsiRNAs.

a. Single-nucleotide resolution maps of RRSV-vsiRNAs in RRSV singly infected rice from the genomic segments S1~S10; b. Single-nucleotide resolution maps of RRSV–vsiRNAs in doubly infected rice from the genomic segments S1~S10; c. Single-nucleotide resolution maps of SRBSDV-vsiRNAs in SRBSDV singly infected rice from the genomic segments S1~S10; d. Single-nucleotide resolution maps of

SRBSDV-vsiRNAs in doubly infected rice from the genomic segments

S1~S10
